# Wound-Healing Potential of Engineered Lysin GRC-ML07 in *Pseudomonas aeruginosa* Infected Wounds in Immunocompromised Mice

**DOI:** 10.3390/antibiotics14121248

**Published:** 2025-12-10

**Authors:** Mariam Abdulkadieva, Darya Slonova, Veronika Litvinenko, Nataliia Antonova, Elena Mazunina, Konstantin Sobyanin, Tatiana Guseva, Olga Parshina, Pavel Domnin, Vladislav Poloskov, Vladimir Guschin, Daria Vasina, Elena Sysolyatina, Alexander Gintsburg

**Affiliations:** 1 Gamaleya Research Center of Epidemiology and Microbiology, Ministry of Health of Russian Federation, Moscow 123098, Russia; 2 Joint Institute of High Temperatures, Russian Academy of Science, Moscow 125412, Russia; 3 Moscow Center for Advanced Studies, Moscow 123592, Russia; 4 Institute of Translational Medicine, Pirogov Russian National Research Medical University, Moscow 117513, Russia; 5 Department of Medical Genetics, Sechenov First Moscow State Medical University, Moscow 119991, Russia; 6 Department of Infectiology and Virology, Sechenov First Moscow State Medical University, Moscow 119991, Russia

**Keywords:** lysin, wound healing, antibacterial effect, *Pseudomonas aeruginosa*, immune suppression, mouse model

## Abstract

**Objectives:** The study aimed to evaluate the antibacterial and wound-healing potential of the engineered lysin GRC-ML07 in a mouse model of full-thickness wounds infected with multidrug-resistant *Pseudomonas aeruginosa* under immunosuppression. **Methods:** Male BALB/c mice (22–24 g) were immunocompromised with cyclophosphamide. Three days later, full-thickness excisional wounds were created and infected with *P. aeruginosa* (10^7^ cells/wound). The lysin GRC-ML07 incapsulated into an alginate gel was applied topically to the wound area twice a day for four days after infection. Wound swabs for microbiological assays and scab tissues for cytokine and cellular profiling were collected on days 4 and 7. Histological samples were taken on days 4, 7, 14, and 21. **Results:** Lysin GRC-ML07 induced bacterial lysis accompanied by low activation of TLR2, TLR4, or TLR7/8 signaling pathways and pro-inflammatory cytokine production *in vitro*. Its application *in vivo* resulted in decreased levels of GM-CSF, IL-1β, IL-6, IL-17A, and TNF-α in the wound, accompanied by a 46% increase in neutrophil counts on day 4 compared to control and placebo (alginate gel) groups. By day 7, lysin treatment reduced bacterial load by 2 log, decreased neutrophil counts in wounds, and led a transition of the wounds to the granulation and epithelialization phase with scab desquamation. **Conclusions:** It was first shown that engineered lysin GRC-ML07 exhibits not only antibacterial, but pronounced pro-healing effects in immunocompromised mice, promoting resolution of inflammation and transition to the granulation/epithelialization phase.

## 1. Introduction

Wound healing is a fundamental physiological process essential for maintaining skin integrity. Impaired healing often results in chronic wounds and hypertrophic scars and keloid formation. Wound infection aggravates the pathological process, prolongs treatment duration, increases the risk of disability, and, in severe cases, leads to death [1,2].

The number of people with secondary immune suppression increases in the developed countries due to advances in the management of viral diseases (HIV), hematologic malignancies, solid organ and bone marrow transplantation [3] and also due to an increased life expectancy. A treatment of immunocompromised patients with wounds remains a major clinical challenge because they have an increased susceptibility to microbial infections. Immunosuppression can be induced not only by pathogens, drugs, tumor process, hormone imbalance, but by different etiological stress, severe traumas and surgical interventions [4]. Post-operative and post-traumatic immune suppression can last from one to four weeks, with a peak typically observed on day two after surgery [5,6,7]. One of the mechanisms underlying immunosuppression in such patients involves activation of the hypothalamus–pituitary–adrenal axis, enhanced secretion of adrenocorticotropic hormone, and increased plasma glucocorticoid concentrations. These processes inhibit inflammatory responses. They induce degradation and apoptosis of T lymphocytes (especially T helper cells) and eosinophils. In addition, they impair the migration of lymphocytes from peripheral lymphoid organs such as the lymph nodes, spleen, and thymus [8]. Hospital patients are also at higher risk of being infected by multidrug-resistant wound contaminants—*Staphylococcus aureus* and *Pseudomonas aeruginosa* [9,10]. So, the development of new antibacterial therapeutic agents for infected wound treatment of immunosuppressed patients is of high relevance.

Enzibiotics are a promising class of antimicrobial agents. The term “enzibiotics” originates from the combination of “enzymes” and “antibiotics.” Although the literature most often refers to enzymes derived from bacteriophage genomes—particularly lysins—the range of proteins considered as potential antibacterial molecules is much broader [11,12,13].

Lysins exhibit high species specificity, minimal or no cytotoxicity toward eukaryotic cells, and a limited impact on the host microbiota [14]. Development of bacterial resistance to lysins is considered unlikely for the following reasons: (i) lysins interact with highly conserved cell wall components; and (ii) bacteriophages and bacteria have co-evolved for millions of years. Although the activity of lysins against Gram-negative bacteria is often hampered by the outer membrane barrier, several lysins active against ESKAPE pathogens (*Enterococcus faecium*, *Staphylococcus aureus*, *Klebsiella pneumoniae*, *Acinetobacter baumannii*, *Pseudomonas aeruginosa*, and *Enterobacter* spp.) have been characterized [15]. Moreover, genetic engineering approaches such as fusion with membrane-permeabilizing antimicrobial peptides have been used to enhance their efficacy against Gram-negative species [16].

Another advantage of lysins is their antibiofilm activity. Incubation of engineered lysins with biofilms of *Klebsiella pneumoniae* (rich in extracellular polysaccharides) and *Acinetobacter baumannii* (poor in matrix) resulted in at least a twofold reduction in biofilm biomass. This effect was attributed to the degradation of acidic exopolysaccharides (as shown for LysAp22), strong DNA-binding capacity (LysAm24), or a combination of both mechanisms [17]. In other studies, an alginate-based gel formulation containing the lysin LysSi3 and lysostaphin inhibited the growth of *Staphylococcus aureus*, *Pseudomonas aeruginosa*, and *Klebsiella pneumoniae* on skin, as well as the formation of mixed-species biofilms, through matrix disruption [18].

Our previous *in vivo* studies have demonstrated the therapeutic potential of engineered lysin-based enzibiotics [19]. The candidate enzyme LysECD7-SMAP showed efficacy in the treatment of systemic *Klebsiella* infections and local *Pseudomonas* infections associated with chronic wounds (trophic ulcers). The developed formulations significantly reduced animal mortality during bacteremia and enabled complete re-epithelialization by day 14 post-infection in all animals of the experimental group. Despite a substantial body of work on the use of lytic enzymes for antibacterial therapy both *in vitro* and *in vivo*, the precise mechanisms of enzibiotic action remain incompletely understood. Furthermore, there is no evidence that these medicines can be effective in immunosuppressed patients.

Therefore, the aim of this study was to assess the wound-healing potential of the engineered lysin GRC-ML07 in *Pseudomonas aeruginosa* infected wounds in immunocompromised mice.

## 2. Results

### 2.1. In Vitro Antibacterial, Immunomodulatory and Cytotoxic Activity of Lysin GRC-ML07

**Figure 1:**
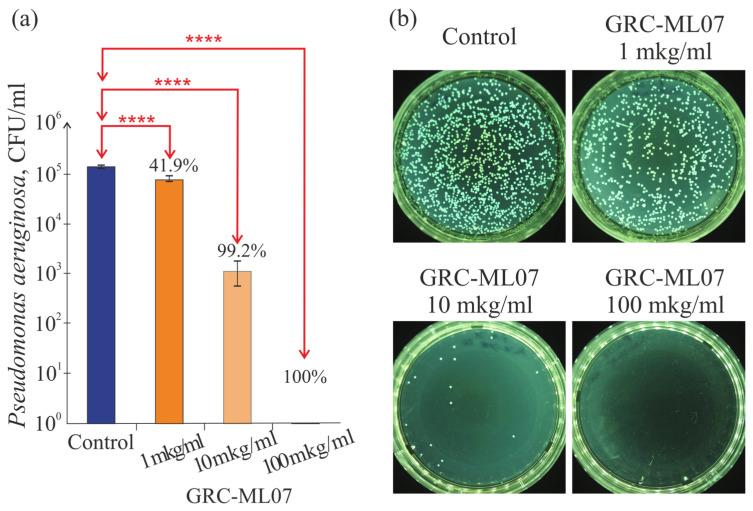
Dose-dependent antibacterial effect of GRC-ML07 against *P. aeruginosa* 21 *in vitro*. CFU counts (**a**) and plates photos (**b**) of *P. aeruginosa* after incubation with different concentrations of lysin. The activity was assessed in triplicate in three independent experiment. ****—*p* < 0.05.

Recombinant lysin GRC-ML07 demonstrated pronounced dose-dependent antibacterial activity against *Pseudomonas aeruginosa* strain 21 *in vitro*. In a plate test enzyme exhibited detectable lytic effects at concentrations as low as 1 μg/mL, while treatment at 100 μg/mL resulted in up to a 10^5^-fold reduction in viable bacterial counts (Figure 1).

To evaluate potential immunomodulatory properties of the lysin GRC-ML07 itself and products of bacterial lysis after *P. aeruginosa* treatment with the enzyme, the THP1-Dual™ reporter cell line was used. This system enables simultaneous assessment of the NF-κB and interferon regulatory factors (IRFs) signaling pathways in human monocytes.

**Figure 2:**
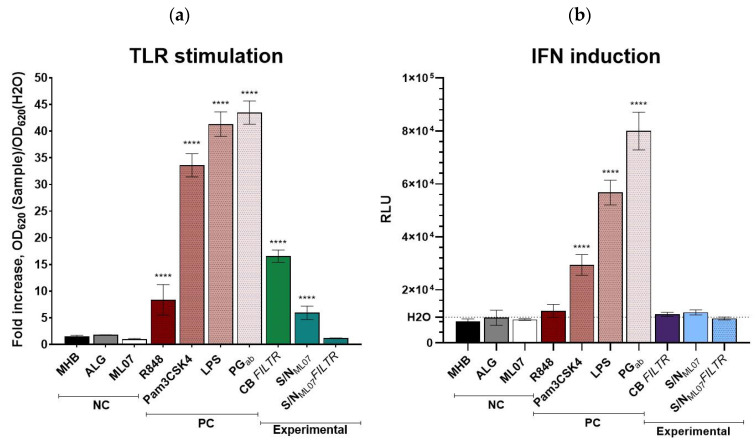
Activation of NF-κB (**a**) and IRF (**b**) signaling pathways in THP1-Dual™ monocytes by alginate gel components, GRC-ML07, and bacterial lysates. NC—negative control; PC—positive control. Dashed line indicates luciferase activity in water-treated cells. RLU—relative light units. MHB (NC)—Mueller–Hinton broth sterile; Alg—Sodium alginate, 1%; ML07—GRC-ML07; R848 (PC)—TLR7/8 ligand; Pam3CSK4 (PC)—Synthetic triacylated lipopeptide and a TLR2/TLR1 ligand; LPS (PC)—Purified lipopolysaccharide from *E. coli* strain 055:B5. TLR4 ligand; PG*ab*—Bacterial peptidoglycan. TLR2 ligand; CB FILTR—Filter-sterilized culture broth after intact *P. aeruginosa* cells cultivation; S/N_ML07_—Supernatant of *P. aeruginosa* after the treatment with 1 mg/mL of GRC-ML07; S/N_ML07_ FILTR—Filter-sterilized supernatant of *P. aeruginosa* after the treatment with 1 mg/mL of GRC-ML07. All experimental variants were performed in triplicate in two independent experiments. ****—*p* < 0.0001 compared to MHB stimulated samples.

As expected, strong activation of both pathways was observed in response to canonical TLR ligands: *A. baumannii* peptidoglycan and *E. coli* LPS induced NF-κB activity 43.5- and 41.3-fold and IFN pathway activation 8.2- and 5.8-fold above the negative control (water), respectively. The synthetic triacylated lipopeptide Pam3CSK4 increased NF-κB and IFN activity 8.4- and 3.0-fold, whereas R848, a TLR7/8 agonist, selectively triggered NF-κB activation (8.4-fold) (Figure 2).

Among the tested samples, neither alginate gel nor GRC-ML07 alone significantly induced the IRF pathway. However, modest activation of NF-κB signaling was observed, particularly in response to the filter-sterilized culture broth of *P. aeruginosa* 21, which increased NF-κB and IRF activities 16.6-fold compared to control. This suggests the presence of soluble pathogen-associated molecular patterns (PAMPs) retained in the cell-free filtrate. The average concentration of viable cells in all stimulation variants ranged 4.9–5.4 × 10^5^ cells/mL (98,900–108,600 cells/well), with 5.1–5.2 × 10^5^ cells/mL (102,400–103,900 cells/well) for the NC controls (H_2_O and MHB) (Figure S1). This indicates comparable counts of viable cells in all the samples studied. The use of alginate gel components, GRC-ML07, and bacterial lysates did not significantly affect the viability of THP-1 cells, while the incubation of samples with *P. aeruginosa* resulted in the monocyte’s death.

Notably, the lysate of bacterial cells treated with GRC-ML07 also induced NF-κB signaling (~6-fold), but this effect was abolished after filtration through a 0.22 μm membrane. We hypothesize that this may result from the binding properties of the lysin molecule. Our previous studies demonstrated that bacteria exposed to native or modified lysins form massive aggregates visible under scanning electron microscopy [20], and that these enzymes can tightly interact with bacterial components such as peptidoglycan, lipopolysaccharides (LPS), and DNA through electrostatic interactions [17]. Thus, we suppose PAMPs to be bound within these aggregates and be retained on the filter membrane during sterilization, leading to decreased TLR-stimulating activity of the filtrates. A comparable phenomenon has been reported for polymyxins, whose bactericidal activity involves displacement of divalent cations (Ca^2+^, Mg^2+^) from LPS, accompanied by endotoxin neutralization [21]. Importantly, all supernatants, regardless the filtration step, were confirmed to be sterile, indicating that the observed responses were not due to bacterial contamination or metabolite production.

Taken together, these data suggest that treatment of *P. aeruginosa* with GRC-ML07 leads to the release of limited amounts of potential TLR ligands, such as TLR2/TLR1 activators (e.g., peptidoglycan) [22], TLR7/TLR8 agonists (nucleotides or ssRNA) [23,24], and TLR4 agonists (LPS) [25]. However, their concentration in the post-lysis supernatants is insufficient to significantly stimulate IRF-dependent type I interferon production in THP1 monocytes, resulting instead in a moderate pro-inflammatory NF-κB response.

**Figure 3:**
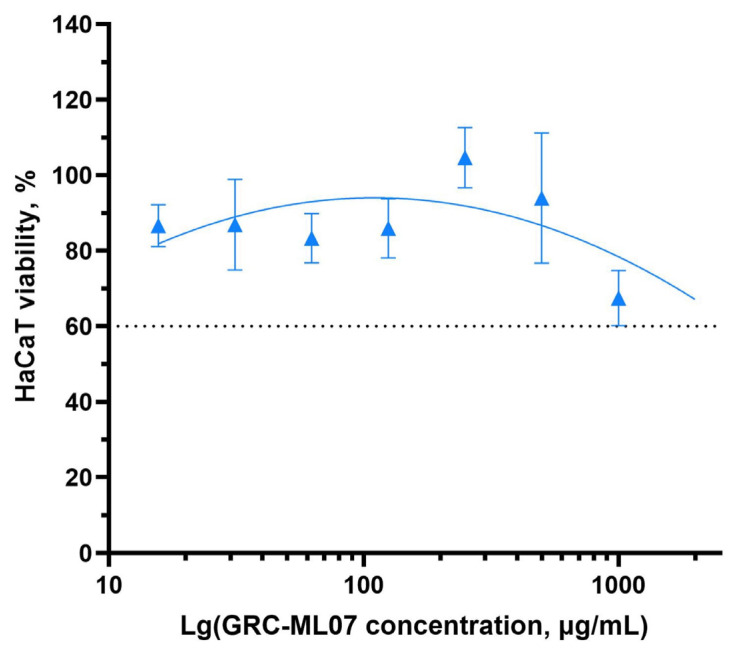
Effect of GRC-ML07 on the viability of HaCaT keratinocytes assessed by MTT assay after 24 h incubation with concentrations ranging from 15.6 µg/mL to 1000 µg/mL. Data represent mean ± SD of three independent experiments. The threshold of significant viability reduction is indicated with a dashed line.

Previous preclinical studies have demonstrated the safety of endolysin-based therapeutics following both topical and systemic administration, supporting the absence of adverse systemic effects associated with this class of antimicrobial agents [26,27]. We evaluated the effect of GRC-ML07 on the viability of human keratinocytes using the HaCaT cell line and an MTT assay (Figure 3). GRC-ML07 exhibited moderate cytotoxicity *in vitro*, most pronounced at the highest tested concentration of 1000 µg/mL, at which cell death did not exceed 60%.

### 2.2. Antibacterial and Wound Healing Effect of Topical Applications of GRC-ML07

**Figure 4:**
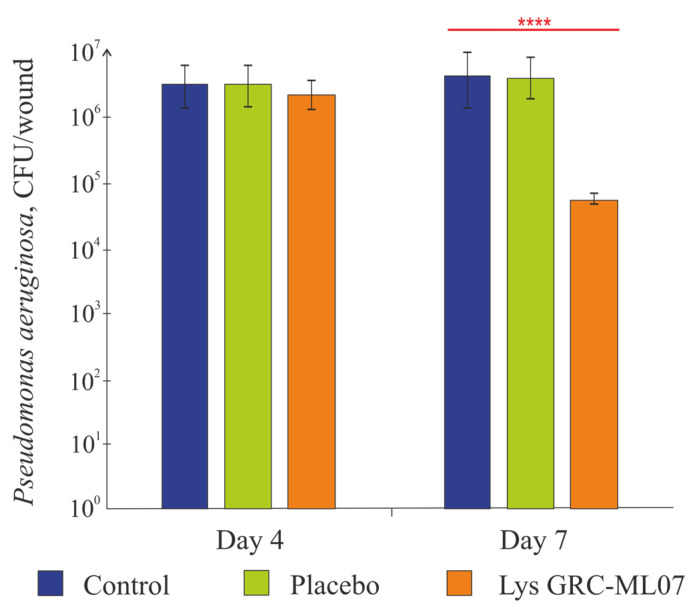
Wound bacteria reduction on the 4th and 7th days of observation. ****—*p* < 0.05. Data represent mean ± SD of three independent experiments.

Topical administration of GRC-ML07 significantly reduced bacterial colonization of the wound in immunosuppressed mice. According to microbiological data (Figure 4), the alginate gel used as placebo did not affect bacterial load. While no statistically significant antibacterial effect was detected after four topical applications (day 4 post-infection), by day 7 (after 8 applications), bacterial counts in the GRC-ML07–treated group decreased by 2 log units compared to control or placebo groups (*p* < 0.05).

**Figure 5:**
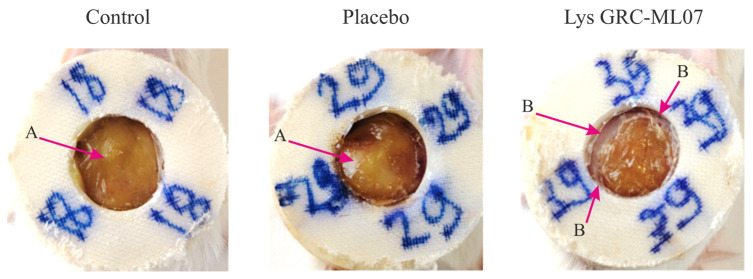
Macroscopic changes in the condition of wounds on the 7th day of observation. A—purulent masses, B—scab desquamation.

Animals treated with lysin demonstrated earlier desquamation of the wound scab. Although the *in vivo* antibacterial effect of GRC-ML07 was lower than its *in vitro* efficacy, macroscopic differences in the wound bed appearance were pronounced. By day 7, the control and placebo groups showed accumulation of purulent exudate beneath the scab, whereas the wounds treated with lysin appeared dry with peripheral scab detachment (Figure 5).

### 2.3. Lysin GCR-ML07 Effects to Re-Epithelization and Tissue Remodeling

**Figure 6:**
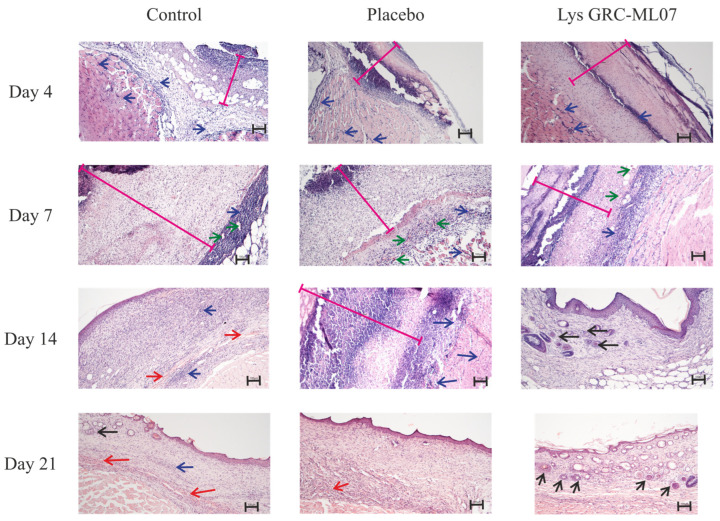
Histological analysis of scab tissue on the 4th, 7th, 14th and 21st days. Pink line—purulent-necrotic debris; red arrow—diapedetic hemorrhages; green arrow—neovascularization/blood vessel; blue arrow—leukocytic infiltration; black arrow—folliculogenesis. The division value on the scale is 100 µm.

Histological analysis revealed that by day 4 post-infection, no differences were observed among control, placebo, and lysin-treated groups (Figure 6). In all samples, the wound defect was covered by abundant purulent-necrotic material overlying fibrin deposits densely infiltrated by leukocytes and focal diapedetic hemorrhages. The subcutaneous loose connective tissue was infiltrated with neutrophils, and sparse fibroblast migration was evident. Early signs of neoangiogenesis were present, but most vessels had empty lumens. Skeletal muscle showed vascular congestion, leukostasis, and perivascular infiltration consistent with acute myositis.

By day 7, distinct histological differences emerged. In the control and placebo groups, the wound defect remained covered with compact fibrin and disintegrated leukocytes. Granulation tissue formation was evident beneath the scab, with congested vessels, ongoing angiogenesis, fibroblast migration, and moderate leukocyte infiltration. Signs of inflammation persisted, including leukostasis and marginal leukocyte adhesion within vessel lumens.

In contrast, wounds treated with GRC-ML07 exhibited earlier epithelial migration and organized granulation tissue. A moderate fibrin layer containing leukocytes overlayed a dense inflammatory infiltrate. Beneath this layer, a continuous zone of granulation tissue was observed, with pronounced fibroblast proliferation, active angiogenesis, and partial epithelial ingrowth from the wound margins.

On day 14, GRC-ML07–treated wounds were fully re-epithelialized with keratinization of the superficial layer, folliculogenesis, and hair growth near the wound margins. Mature granulation tissue underwent reorganization into loose connective tissue. In control and placebo groups, however, strong lymphohistiocytic infiltration persisted within granulation tissue and subcutaneous muscle, accompanied by focal hemorrhages. Early epithelial closure was observed only in some animals.

By day 21, uniform folliculogenesis across nearly the entire wound area and desquamation of the keratinized layer were evident in the experimental group, indicating advanced tissue remodeling. Control wounds still displayed focal hemorrhages and only partial keratin desquamation.

Thus, topical application of GRC-ML07 promoted re-epithelialization and earlier remodeling of the granulation tissue into mature connective tissue compared with control and placebo groups.

### 2.4. Local Changes in Cell Components and Cytokine Production Under GRC-ML07 Treatment

Multiplex analysis of scab tissue and flow cytometry were used to assess cytokine and immune-cell profiles. Detected cytokines included GM-CSF, IL-1β, IL-6, IL-10, IL-17A, and TNF-α, whereas IFN-γ, IL-2, IL-4, and IL-5 were below the detection threshold.

**Figure 7:**
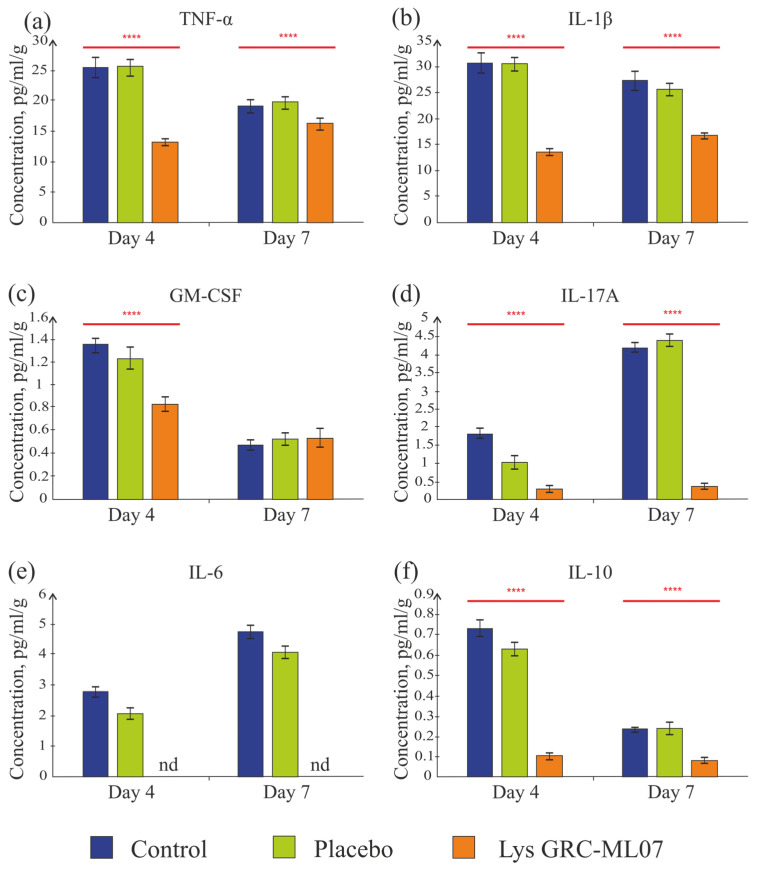
Dynamics of cytokine (TNF-α (**a**), IL-1β (**b**), GM-CSF (**c**), IL-17A (**d**), IL-6 (**e**), IL-10 (**f**)) levels in wounds. nd—not detected. ****—*p* < 0.05. Data represent mean ± SD of three independent experiments.

Despite similar bacterial load and histological characteristics on day 4, lysin-treated animals showed significantly lower levels of key pro-inflammatory cytokines compared to control and placebo groups (Figure 7).

TNF-α, tumor necrosis factor, is a key pro-inflammatory cytokine [28,29]. The concentration of TNF-α in the experimental group was 1.9 times lower than in the control and placebo groups (*p* < 0.05) (Figure 7a). On day 7, the level of TNF-α slightly decreased in all groups compared to day 4—by 1.3 and 1.2 times in the control and placebo groups, respectively. In the group of animals treated with the lysin GRC-ML07, the TNF-α level was 84.3% lower than in the control group (*p* < 0.05). A significant decrease in TNF-α concentration on day 4 could indicate the transition from the inflammatory to the granulation phase of wound healing.

IL-1β regulates local inflammation and contributes to tissue remodeling by activating fibroblasts and keratinocytes, thereby promoting angiogenesis and re-epithelialization. The concentration of IL-1β was 2.3-fold lower in the GRC-ML07 group than in control and placebo on day 4 (Figure 7b). By day 7, the IL-1β levels decreased in all groups, but the difference between the lysin and control groups remained—the concentration in the lysin group was 1.6 times lower than in the control.

GM-CSF (granulocyte-macrophage colony-stimulating factor) stimulates the growth of monocytes, neutrophils, eosinophils, and basophil precursors and activates macrophages. The detectable GM-CSF level was low, but in the experimental group it was still 1.6 times lower on day 4 than in the control and placebo groups. By day 7, all groups showed values slightly above the detection threshold (Figure 7c).

In the lysin-treated group, the detectable IL-17A concentration on days 4 and 7 was slightly above the sensitivity threshold of the system (0.3 pg/mL), while in the control and placebo groups an increase in this cytokine was observed by day 7 (4.2 and 4.4 pg/mL, respectively) (Figure 7d). IL-17 is secreted by the Th17 subpopulation of CD4+ T cells. It is actively involved in immune responses by inducing the synthesis of pro-inflammatory molecules, including chemokines, antimicrobial proteins (AMPs), and matrix metalloproteinases. These molecules are produced by fibroblasts, as well as endothelial and epithelial cells [30]. The low detectable concentration of IL-17A in all groups could be associated with cyclophosphamide-induced immunosuppression. Its absence in the lysin-treated group may indicate a faster progression through the inflammatory phase. This interpretation was later supported by macroscopic and histological findings on day 7.

IL-6 plays a dual role as a cytokine modulating both inflammation and regeneration [31]. During inflammation, it induces monocyte infiltration, IL-4 and IL-13 production by Th2 cells, Th2 and Th17 differentiation, and later the transition from M1 to M2 macrophages and the proliferative phase. In the GRC-ML07 lysin group, this cytokine was undetectable on both day 4 and day 7, whereas in the control and placebo groups, its levels slightly increased to 4.7 and 4.0 pg/mL, respectively (Figure 7e).

The detectable concentration of the anti-inflammatory cytokine IL-10 in scabs ranged from 0.1 to 0.8 pg/mL/g and decreased in the control and placebo groups by day 7, while it remained stable at 0.1 pg/mL/g in the lysine-treated group (Figure 7f). It is possible that different sample preparation protocols are required to detect IL-10. However, it was shown that endogenous IL-10 expression did not alter closure of full thickness excisional wounds if wound hydration and contraction was controlled, as it was [32].

**Figure 8:**
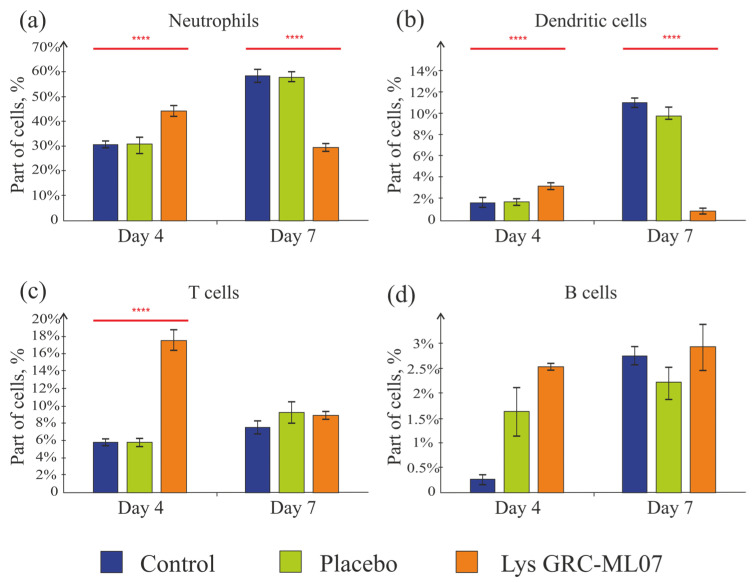
Neutrophils (**a**), dendritic cells (**b**), T and B cells (**c**,**d**) in the scab tissue. **** *p* < 0.05. Data represent mean ± SD of three independent experiments.

Thus, application of lysin GRC-ML07 correlated with a decrease in the levels of pro-inflammatory cytokines in the wound as early as day 4. At the same time, the number of neutrophils in the wounds of this group on day 4 was 46% higher than in the control and placebo. By day 7, in contrast, the control group showed almost a twofold increase in neutrophil numbers, and in the lysin-treated group their number decreased twofold compared to the control (Figure 8a). This may indicate the transition of the wounds to the proliferative/granulation phase by day 7 under lysin therapy.

Dendritic cells were detected in small numbers in the scab tissue in all groups (Figure 8b). This finding is consistent with previously published data, which showed that at the early stages of infection caused by *P. aeruginosa*, there is a local influx of neutrophils. At the same time, dendritic cell subsets become reduced even in immunocompetent mice [33].

T cells are the most predominant lymphocyte subset in human skin wounds; they migrate into the wound and peak during the late proliferative and early remodeling phases [34,35]. According to Huyan X.-H. et al. [36], high doses of cyclophosphamide (100–200 mg/kg) lead to a reduction in the absolute number of lymphocytes on day 1, reaching a minimum on day 4—which corresponds to the day of wound modeling in our experiment. Lymphocyte counts recovered by day 10 (day 6 in our experimental timeline) and then declined again by day 17 (day 13 of our experiment). Moreover, the suppressive effect on B cells was even more pronounced than on T cells. In our study, we also observed low baseline levels of T and B cells in the scab tissue on day 4, with a slight increase by day 7 (Figure 8c,d). In the group treated with lysin GRC-ML07, the number of T cells on day 4 was three times higher than in the placebo and control groups, and by day 7, the values became comparable.

Thus, it was established an increase in neutrophils number on day 4 in the lysin-treated group and their decrease in the scab tissue by day 7 compared to the control and placebo. Dendritic cells, T, and B lymphocytes were detected in small amounts, which did not contradict previously published data.

## 3. Discussion

Wound healing is a complex process involving numerous cells that produce cytokines with pleiotropic effects [37,38,39] and consists of four overlapping phases—hemostasis, inflammation, proliferation, and remodeling. Any disruption of these processes can lead to abnormal wound healing [40]. A key phase of wound healing is the transition from the inflammatory phase to the granulation/epithelialization phase [41]. An impairment of this transition causes to an impairment of wound healing and contributes to chronic wound formation [42].

Infection of wound bed, especially with multidrug-resistant microorganisms, often results in a prolonged inflammation phase and a violation of wound healing [43]. Another risk factor is immunosuppression, which occurs not only in patients with immunodeficiencies or in elderly individuals, but also as a consequence of massive blood loss, severe injuries, surgical interventions, sepsis, and post-traumatic shock [44,45,46].

Promising drugs for the treatment of infections caused by multidrug-resistant bacteria are enzibiotic endolysins. Some of them are effective against Gram-negative bacteria [15,20,47], including multidrug resistant *P. aeruginosa*, one of the most dangerous wound contaminants leading to fatal outcomes [48].

We have shown the potential of the engineered lysin GRC-ML07 to reduce bacterial load and to stimulate the transition of the *P. aeruginosa* infected wound from the inflammatory to the granulation/epithelialization phase using an immunocompromised mouse model. Previously, the effectiveness of a mixture of three engineered lysins (LysAm24, LysAp22, and LysECD7) targeting Gram-negative bacteria was demonstrated in immunocompetent rabbit model of abscess caused by *Fusobacterium necrophorum*. Besides almost doubling the animals lifespan after the infection, the use of the lysin-based antibacterial gel reduced leukocyte and segmented neutrophil counts in the blood indicating its potential to directly or indirectly affect the components of immune system [8,18]. According to our results, the antibacterial effect of topical application of lysin GRC-ML07 in alginate gel caused a 2-log reduction by day 7 of the experiment. These data are consistent with [49,50], but it should be noted that in the mentioned studies, a similar (2 log) antibacterial effect was observed immediately after a single lysin application in wounds of immunocompetent animals. In our work, the antibacterial effect of lysin was evaluated at different time points, which, in our opinion, more accurately reflects the dynamics of the infectious process.

Macroscopically and histologically, lysin GRC-ML07 treatment caused significant changes in the state of the wounds and the transition from the inflammatory to the granulation/epithelialization phase. The very first signs of wound healing acceleration were detected on day 4, after four topical applications of the lysin GRC-ML07. Simultaneously it was also established a decrease in pro-inflammatory cytokine concentrations in the wounds and a 46% increase in neutrophil amount compared to the control groups. This relatively low level of pro-inflammatory cytokines was unexpected, as the destruction of bacterial cells should lead to the formation of PAMPs [51]. PAMPs include carbohydrates such as lipopolysaccharide (LPS) and mannoses; nucleic acids; peptides comprising microtubules and flagellin; and cell wall molecules such as peptidoglycan and lipoteichoic acid [52]. They activate innate immune responses and trigger inflammation accompanied by cytokine and chemokine release, neutrophil and monocyte recruitment, and initiation of adaptive immunity [53,54]. This process is essential for host defense and wound healing [55] but must be tightly regulated to prevent excessive inflammation and tissue damage. According to our *in vivo* results treatment with the GRC-ML07 lysin did not increase the inflammation in the wounds.

In order to find the cause of this phenomenon, we conducted an *in vitro* experiment. Our data indicated that bacterial cell destruction by lysin GRC-ML07 did not lead to an increase in pro-inflammatory cytokine production in the wound. To understand the underlying mechanism, we evaluated the ability of lysates obtained after GRC-ML07 treatment to activate TLRs. Indeed, lysin-mediated bacterial lysis results in the release of wall and cytoplasmic components (LPS, peptidoglycan, nucleic acids) that can serve as TLR ligands and potentially activate immune responses. Although TLR-dependent recognition is highly plastic, we could not detect TLR signaling leading to increased production of type I interferons after bacterial treatment with lysin. While the use of classical ligands of TLR2/TLR1 (Pam3CSK4, peptidoglycan), TLR4 (LPS), TLR7/8 (R848), and sterile filtrates from intact *P. aeruginosa* culture induced the pro-inflammatory transcription factor NF-κB and interferons, the lysates obtained after lysin treatment behaved differently. They activated only NF-κB, while IFN levels remained at control values. The controls included pyrogen-free water and sterile MHB medium. Lysates did not cause significant induction of TLR2/TLR1, TLR7/TLR8, or TLR4, which are responsible for the production of pro-inflammatory cytokines and type I interferons [56,57].

This is consistent with extensive data accumulated for another enzyme, that destructs cell walls—lysozyme. A detailed review [58] describes its immunomodulatory role, showing that bacterial fragments formed after lysozyme exposure exert a lower pro-inflammatory effect than LPS. Furthermore, lysozyme application suppressed TNF-α and IL-6 production by macrophages [59]. Similar results were obtained for a lysin, that was used against *Staphylococcus aureus*. The reduced levels of TNF-α, IL-1β, and IFN-γ were detected in this work [60]. These findings fully correspond to our data, where on day 4 of the experiment we observed a 1.9-fold decrease in TNF-α and a 2.3-fold decrease in IL-6 levels in the GRC-ML07 treatment group.

We also demonstrated that the application of lysin increased the number of neutrophils in the wound bed by day 4, while by day 7 their number was already lower than in the control and placebo groups. Overall, this may indicate the completion of the inflammatory phase and the transition to the granulation phase, which was confirmed histologically. However, the question of the functional activity of neutrophils and macrophages in wounds exposed to lysin requires further investigation. For lysozyme, it has been shown that, on the one hand, similar to macrophages, lysozyme-mediated destruction of phagocytosed bacteria may enhance neutrophil activity [61], while on the other hand, lysozyme-mediated degradation of peptidoglycan can limit phagocyte activation and recruitment [62,63,64]. It can be assumed that the lysin GRC-ML07 treatment of *P. aeruginosa* infected wounds led to the destruction of part of the bacterial population without additional activation of immune cells or excessive production of pro-inflammatory cytokines. The mild antibacterial effect of lysin, which did not provoke excessive inflammation, contributed to a faster resolution of the inflammatory phase and accelerated wound healing in immunocompromised mice.

## 4. Materials and Methods

### 4.1. Bacterial Strain

A multidrug-resistant clinical isolate of *Pseudomonas aeruginosa* (strain 21) from the collection of the N.F. Gamaleya National Research Center for Epidemiology and Microbiology (Moscow, Russia) was used in this study. The strain was resistant to ampicillin, amoxicillin/clavulanic acid, cefazolin, cefotaxime, cefoperazone/sulbactam, imipenem, meropenem, amikacin, gentamicin, netilmicin, nitrofurantoin, and trimethoprim/sulfamethoxazole.

### 4.2. Immunosuppressed Wound Model

Male BALB/c mice (22–24 g; Andreevka breeding facility, NRC “Biotechnology”, FMBA of Russia) were used in the experiments. The use of males is due to the fact that early healing in females occurs faster due to the effect of estrogen on angiogenesis, as well as differences in the structure of the skin of males and females. Immunosuppression was induced by a single intraperitoneal injection of cyclophosphamide at a dose of 1200 µg per mouse (“BELMEDPREPARATY”, Minsk, Belarus) administered four days before wound creation.

For wound creation, mice were shaved (trimmer MOSER Rex Mini 1411-0062, St. Georgen, Germany), depilated (Veet, Reckitt Benckiser, Mississauga, ON, Canada), and the skin surface was degreased with 70% ethanol. Anesthesia was induced using isoflurane delivered via a MultiAnimal gas anesthesia system (BrainTree, Chicago, IL, USA). Once anesthesia was achieved, full-thickness excisional wounds (12 mm in diameter) were created on the dorsum using a sterile biopsy punch (FASTER TOOLS FT293-03, Shanghai, China).

To prevent wound contraction, the wound edges were fixed with an adhesive ring (Extra Soft, Lucca, Italy) containing a central perforation matching the wound diameter (12 mm). Each wound was inoculated with 100 µL of a bacterial suspension containing *P. aeruginosa* strain 21 (10^7^ cells/mouse) prepared in 1.5% methylcellulose gel, followed by sealing with a transparent medical film.

The treatment regimen included three groups:

Control—infected, untreated;
Placebo—infected, treated with 100 µL alginate gel;
Lysin—infected, treated with 100 µL of lysin GRC-ML07 (1 mg/mL in alginate gel).

Topical applications were performed twice a day for four days following infection: the 1st application was made 24 h post wound creation. Animal condition and wound appearance were monitored daily. On days 4 and 7 post-infection, wound surface wash samples were collected for microbiological culture, and scab tissue was harvested for analysis of the immunological and cytokine profiles of the wound bed. For histological examination, tissue samples were collected on days 4, 7, 14, and 21 post-infection.

### 4.3. Production and Purification of Lysin GRC-ML07

Recombinant lysin GRC-ML07 was produced as described previously [65] using *E. coli* BL21(DE3)pLysS for heterologous expression. Two-step purification included cation-exchange chromatography on SP-Sepharose resin (GE Healthcare, Chicago, IL, USA) followed by size-exclusion chromatography on a Superdex 75 pg column (GE Healthcare, USA). The purified protein was eluted with phosphate-buffered saline (PBS, VWR, Radnor, PA, USA), lyophilized (FreeZone, Labconco, Kansas City, MO, USA), and stored at −80 °C until use. Protein concentration was determined spectrophotometrically at 280 nm (NanoPhotometer, IMPLEN, Munich, Germany) using a theoretical extinction coefficient of 0.852, and purity was confirmed by 16% SDS-PAGE.

### 4.4. Gel Formulation with Lysin GRC-ML07

A 2% solution of algae sodium alginate (AppliChem, Darmstadt, Germany) in a sterile phosphate saline buffer (PBS pH 7.4) was prepared by mixing until complete dissolution and was sterilized by autoclaving. After gel base cooling, filter-sterilized GRC-ML07 (1 mg/mL final concentration in enzyme gel) or PBS pH 7.4 (1% alginate vehicle) were aseptically added in equal proportions and mixed thoroughly.

### 4.5. In Vitro Antibacterial Activity Assay

An overnight culture of *P. aeruginosa* strain 21 was diluted in Mueller–Hinton broth (MHB, HiMedia Laboratories Pvt. Ltd., Mumbai, India) to OD_600_ = 0.6, centrifuged (6000× *g*, 5 min), and resuspended in PBS to yield 10^8^ CFU/mL (0.5 McFarland standard). The suspension was further diluted to 10^6^ CFU/mL. 100 µL of bacterial suspension was mixed with 100 µL of the test sample (GRC-ML07) or PBS (control) in sterile 96-well plates. Mixtures were incubated for 30 min at 37 °C with shaking (200 rpm), serially diluted, plated on Mueller–Hinton agar (MHA), and incubated for 16–18 h at 37 °C. The colony-forming units (CFU) were counted, and antibacterial activity (*X*, %) was calculated as:

$$X=100\%-\;\frac{{CFU}_{sample}\;\times \;100\%}{{CFU}_{control}},$$

${CFU}_{sample}$—the number of colony-forming units in the test sample, CFU/mL;

${CFU}_{control}$—the number of colony-forming units in the control sample, CFU/mL.

The activity was assessed in triplicate in three independent experiment.

### 4.6. Study of the Effect of Gel Components on NF-kB and IRF Signaling Pathways of Monocytes In Vitro (THP1-Dual™ Reporter Assay)

THP1-Dual™ cells (Invivogen, San Diego, CA, USA) were used for analysis. This cell line was engineered from human THP-1 monocytes to express two inducible reporter genes, SEAP to measure NF-κB activation and Lucia luciferase to measure IRF3 activation.

Samples for testing, including positive (PC) and negative (NC) controls were prepared as follows (Table 1). Overnight bacterial cultures of *P. aeruginosa* 21 (OD_600_ of 1.4–1.6) were grown in MHB and centrifuged at 6000× *g* for 10 min, diluted with fresh medium and grown for 2 h to the exponential phase (OD_600_ of 0.6).

The sterile Mueller–Hinton broth was collected on this stage from the same media stock. After two hours, cells were harvested and samples of media after intact *P. aeruginosa cultivation* (CB FILTR) were collected. Bacterial cells were further treated with the lysin in concentration 1 mg/mL as described above (S/N_ML07_) or alternatively disrupted with sonication (S/N_us_). Treated samples were partly filter-sterilized for analysis (S/N_ML07_ FILTR and S/N_us_ FILTR). 100 μL of each sample before and after the sterilization were plated onto an MH agar and examined after an overnight incubation at 37 °C to confirm absence of viable bacteria colonies.

Prior to the assay, THP1-Dual™ cells were transferred to a T75 flask at a density of 3.0 × 10^6^ and were cultured in 15 mL of RPMI 1640 (Capricorn) containing 10% heat-inactivated (30 min at 56 °C) fetal bovine serum (Citiva, Marlborough, MA, USA), 2 mM Glutamin (Capricorn, Ebsdorfergrund, Germany), 25 mM HEPES (GIBCO, Waltham, MA, USA), and Penicillin-Streptomycin (100 U/mL–100 μg/mL; GIBCO). They were passaged after reaching densities of 1–2 × 10^6^ cells/mL. After that cells were collected by centrifugation (350× *g*, 10 min, RT), washed in PBS. For analysis cells were resuspended at 50,000 cells per well in 0.18 mL freshly prepared test medium in a standard 96-well tissue culture-treated plates (Corning, Corning, NY, USA) and 20 μL of tested compounds were added. The plates were then incubated in a humidified chamber at 37 °C and 5% CO_2_ for 20 h. All experimental variants were performed in triplicate.

After incubation, 20 μL of the supernatants were used to determine the SEAP levels with QUANTI-BlueTM Solution (InvivoGen, USA) during 3 h of incubation as per manufacturer’s protocol in the Thermo fisher reader (Waltham, MA, USA). And 20 μL of the supernatants were used to determine luciferase activity with the QUANTI-LucTM 4 reagent (InvivoGen, USA). Readings were counted in relative luminescence units (RLU) for luciferase and as OD_620_ fold change to control with water for *SEAP*:

$$SEAP\;level\;=\;\frac{{OD}_{620}sample}{{OD}_{620}H_{2}O}$$

For quantitation of cell viability after the incubation with tested compounds, 20 μL of cells suspension were mixed with 20 μL of 0.4% *v*/*v* trypan blue stain (Bio-Rad, Hercules, CA, USA) and examined using TC20 Automated Cell Counter (Bio-Rad, Hercules, CA, USA) (Figure S1).

### 4.7. HaCaT Cytotoxicity Assessment

The cytotoxic properties were evaluated for HaCaT (ATCC PCS-200-011) cell lines by MTT assay. For analysis HaCaT cells (1 × 10^4^ cells/well) were added to 96-well plates and were cultivated in 100 µL of the full growth medium (DMEM/F12 (Capricorn Scientific, Ebsdorfergrund, Germany) supplemented with 10% FBS (Sigma-Aldrich, Burlington, MA, USA) and 2 mM L-glutamine (Paneco, Moscow, Russia)) over 24 h at 37 °C in a humidified atmosphere with 5% CO_2_, until 80–90% of confluent monolayers were achieved. After incubation, the medium was removed and 100 µL of GRC-ML07 serial dilutions in the growth medium starting at 1000 µg/µL were added into each well. Cells cultivated in 100 µL of the growth medium were used as a negative control with 100% cell viability, while cells incubated with 0.1% Triton X-100 (SERVA Electrophoresis GmbH, Heidelberg, Germany) solution were used as a positive control with a 0% cell viability. Each sample type was analyzed in three replicates for statistical sampling.

The plates were incubated (37 °C, 5% CO_2_) for 24 h and the cells were then stained with 10% MTT (neoFroxx GmbH, Einhausen, Germany) for 4 h. After incubation, the wells content was replaced by 100 µL of DMSO (Merck, Darmstadt, Germany) and the absorbance at 570 nm (OD_570_) was measured using SPECTROstar NANO (BMG LABTECH, Ortenberg, Germany).

Cell viability was calculated using the following equation:

$$Survival\;\left(\%\right)=\;\frac{ODexp-ODmin}{ODmax-ODmin}*100$$

where *ODexp*—value of *OD*_570_ in the experimental culture wells, a.u.; *ODmax*—average value of the *OD*_570_ of the negative control, a.u.; *ODmin*—average value of the *OD*_570_ of the positive control, a.u.

The experiment was performed in triplicate.

### 4.8. In Vivo Antibacterial Activity Assay

Wound bacterial burden was assessed on days 4 and 7 post-infection. Wound swabs were collected with sterile cotton buds, serially ten-fold diluted in sterile PBS, and plated on Brain Heart Infusion (BHI) agar (Difco, Saint-Ferréol, France). Plates were incubated for 24 h at 37 °C, afterwards colony-forming units (CFU) per wound were calculated.

### 4.9. Necropsy and Sample Collection

Scabs and underlying tissue samples were collected on days 4, 7, 14, and 21. Mice were euthanized using isoflurane anesthesia. Full-thickness skin sections were excised along the wound perimeter, occasionally including underlying vertebrae to prevent deformation of the wound bed during sampling.

### 4.10. Histological Analysis

Excised tissues were fixed in 10% neutral-buffered formalin (pH 7.2–7.4) at room temperature for at least 48 h. A fresh fixative was added after 24 h. After fixation, tissue samples were paraffin-embedded, sectioned, deparaffinized in three lots of xylene, rehydrated through graded ethanol series, and stained with Mayer’s hematoxylin and aqueous eosin (Biovitrum, Saint Petersburg, Russia). Stained sections were dehydrated, cleared in xylene, and mounted using Consul-Mount synthetic mounting medium (Thermo Fisher Scientific, USA) for microscopy.

### 4.11. Cytokine Multiplex Assay

Scabs and underlying tissues were snap-frozen in liquid nitrogen and stored at −80 °C until protein extraction. Frozen tissue (~0.5 g) was homogenized in 300 µL lysis buffer (100 mM Tris-HCl, pH 7.4; 150 mM NaCl; 1 mM EGTA; 1 mM EDTA; 1% Triton X-100; phosphatase and protease inhibitor cocktails). Lysates were incubated at 4 °C for 2 h, centrifuged at 15,000–17,000 g for 20 min at 4 °C, and supernatants collected on ice. Cytokine levels were quantified using Bio-Plex Pro™ Mouse Cytokine Th17 Panel A 6-Plex and Bio-Plex Pro™ Mouse Cytokine Th1/Th2 Assay kits (Bio-Rad), following the manufacturer’s instructions. Cytokine concentrations were normalized to tissue mass.

### 4.12. Flow Cytometry Analysis

Scabs and underlying tissue were collected into individual tubes containing 1 mL PBS with 10 mg/mL gentamicin (Sigma-Aldrich, USA) and incubated at room temperature for 30 min with vortexing. Tissue fragments (~0.5 g) were digested overnight at 4 °C in 1 mL HBSS (Hanks’ Balanced Salt Solution) containing 1 mg/mL dispase (Gibco, USA), 3% FBS (Gibco, USA), and 10 mg/mL gentamicin. Samples were further minced with a sterile scalpel and incubated in 1 mL HBSS containing 1 mg/mL collagenase D (Roche, Basel, Switzerland) and 10 mg/mL gentamicin for 2 h at 37 °C. Digested suspensions were combined with the initial HBSS, centrifuged at 1200 rpm for 10 min at 4 °C, and resuspended in 1 mL PBS (Sigma-Aldrich, USA).

Cells were stained with fluorochrome-conjugated monoclonal antibodies against CD45 (Clone 30-F11), CD11b (Clone M1/70), CD11c (Clone HL3), Ly6G (Clone 1A8), I-A/I-E (Clone M5/114.15.2), CD3 (Clone 17A2), and CD19 (Clone 1D3) (BD Biosciences, Franklin Lakes, NJ, USA) diluted 1:50 for 30 min at 4 °C in the dark. Red blood cells were lysed using RBC lysis buffer (155 mM NH_4_Cl, 10 mM KHCO_3_, 0.1 mM EDTA), washed with PBS, and analyzed on a CytoFLEX flow cytometer (Beckman Coulter, Brea, CA, USA). Isotype controls APC IgG2a k (Clone R35-95), BB700 IgG1 k (Clone A19-3), BV605 IgG2b k (Clone R35-38), BV786 IgG2a k (Clone R35-95), FITC IgG2b k (Clone A95-1) and V450 IgG2b k (Clone A95-1) were used. Flow cytometry acquisition was set to collect 80,000–150,000 events per sample.

### 4.13. Statistical Analysis

All experiments were performed in at least triplicate. Data are presented as mean ± standard deviation (SD). Statistical analysis was conducted using Student’s *t*-test and one-way ANOVA with Tukey’s post hoc test, where appropriate. Homogeneity of variance was assessed using Levene’s test. Differences were considered statistically significant at *p* < 0.05.

All animal procedures were approved by the Institutional Animal Ethics Committee of the N.F. Gamaleya National Research Center for Epidemiology and Microbiology (Protocol No. 67, 23 November 2023) and were performed in accordance with national regulations for laboratory animal care.

## 5. Conclusions

This study established an immunosuppressed mouse model of *Pseudomonas aeruginosa*-infected wounds and demonstrated its suitability for assessing host–pathogen interactions and therapeutic interventions. Like all animal models, this system cannot fully recapitulate the complexity of human wound infections. Although mice provide a genetically tractable and cost-effective platform, important differences in skin structure and wound healing dynamics persist, including the predominance of contraction over re-epithelization. We attempted to mitigate this limitation by using anti-contraction rings around the wound perimeter. Similarly, the use of a single *P. aeruginosa* strain and a specific inoculum does not reflect the genetic and phenotypic diversity of clinical isolates. Cyclophosphamide-induced immunosuppression provides a reproducible means of reducing host defenses; however, it represents only one mechanism of immunodeficiency and does not fully reflect clinically significant conditions such as diabetes, old age, or chronic systemic inflammation. Furthermore, higher infectious doses can be lethal in immunosuppressed animals, requiring careful dose optimization and monitoring.

Within these limitations, our study allows us to draw several important conclusions. First, we demonstrated for the first time that the modified lysine GRC-ML07 not only exhibits potent antibacterial activity but also exhibits significant wound-healing properties. Specifically, (i) GRC-ML07 reduced the bacterial load in *P. aeruginosa*-infected wounds in immunosuppressed mice by approximately 2 logarithms by day 7; (ii) four topical applications of GRC-ML07 reduced levels of key pro-inflammatory cytokines and increased neutrophil counts in wound tissue; (iii) GRC-ML07 treatment promoted the transition from the inflammatory phase to granulation and epithelialization; and (iv) GRC-ML07 induced direct bacterial lysis without significant activation of TLR2, TLR4, TLR7/8, or excessive cytokine release.

Taken together, these results characterize GRC-ML07 as a promising dual-action therapeutic agent capable of reducing pathogen load while simultaneously promoting inflammation resolution and accelerating wound healing. Future studies will focus on elucidating the molecular mechanisms underlying the wound-healing properties of lysins and expanding translational evaluation in more clinically relevant models.

## 6. Patents

The work contains data included in a patent of N.F. Gamaleya National Research Centre for Epidemiology and Microbiology (RU 2813626 C1) related to GRC-ML07 production and *in vitro* antibacterial activity and the authors (N.A., D.V., V.G and A.G.) are listed as the inventors.

## Figures and Tables

**Table 1:** Cells stimulation variants.

	Sample	Description	Method of Sterilization
1	H_2_O (NC)	Endotoxin free water (Charles River Laboratories, Wilmington, MA, USA)	Sterile
2	MHB (NC)	Mueller–Hinton broth sterile (WVR, Radnor, PA, USA)	Autoclavation
3	Alg	Sodium alginate, 1% (Applichem)	Autoclavation
4	ML07	GRC-ML07, 1 mg/mL	0.22 μm
5	Pam3CSK4 (PC)	Synthetic triacylated lipopeptide and a TLR2/TLR1 ligand (Invivogen)	Sterile
6	LPS (PC)	Purified lipopolysaccharide from *E. coli* strain 055:B5. TLR4 ligand (Charles River Laboratories).	Sterile
7	R848 (PC)	TLR7/8 ligand (Invivogen)	Sterile
8	CB FILTR	Filter-sterilized culture broth after intact *P. aeruginosa* cells cultivation	0.22 μm
9	S/N_us_	Supernatant of *P. aeruginosa* after ultrasonic disintegration	No
10	S/N_us_ FILTR	Filter-sterilized supernatant of *P. aeruginosa* after ultrasonic disintegration	0.22 μm
11	S/N_ML07_	Supernatant of *P. aeruginosa* after the treatment with 1 mg/mL of GRC-ML07	No
12	S/N_ML07_ FILTR	Filter-sterilized supernatant of *P. aeruginosa* after the treatment with 1 mg/mL of GRC-ML07	0.22 μm

## Data Availability

The original contributions presented in this study are included in the article/Supplementary Material. Further inquiries can be directed to the corresponding author.

## Supplementary Materials

The following supporting information can be downloaded at: https://www.mdpi.com/article/10.3390/antibiotics14121248/s1, Figure S1. The amount of alive THP-1-Dual™ monocytes after the incubation with tested compounds.

## Abbreviations

The following abbreviations are used in this manuscript:

EDTA	Ethylenediaminetetraacetic acid
GM-CSF	Granulocyte-macrophage colony-stimulating factor
HIV	Human immunodeficiency virus
IFN	Interferon
IFN-γ	Interferon gamma
IL-10	Interleukin 10
IL-17A	Interleukin 17A
IL-1β	Interleukin 1β
IL-2	Interleukin 2
IL-4	Interleukin 4
IL-5	Interleukin 5
IL-6	Interleukin 6
IRFs	Interferon regulatory factors
LPS	Lipopolysaccharides
MHB	Mueller–Hinton broth
NF-κB	Nuclear factor kappa-light-chain-enhancer of activated B cells
RLU	Relative light units
TLR	Toll-like receptor
TNF-α	Tumor necrosis factor alpha
