# Supplementary material for: Wound-Healing Potential of Engineered Lysin GRC-ML07 in Pseudomonas aeruginosa Infected Wounds in Immunocompromised Mice
Source: Antibiotics (Basel). 2025 Dec 10;14(12):1248. doi: 10.3390/antibiotics14121248 (PMC12729281; doi:10.3390/antibiotics14121248)
Supplement: Supplementary file 1 [file antibiotics-14-01248-s001.zip › antibiotics-3981232-supplementary.pdf]

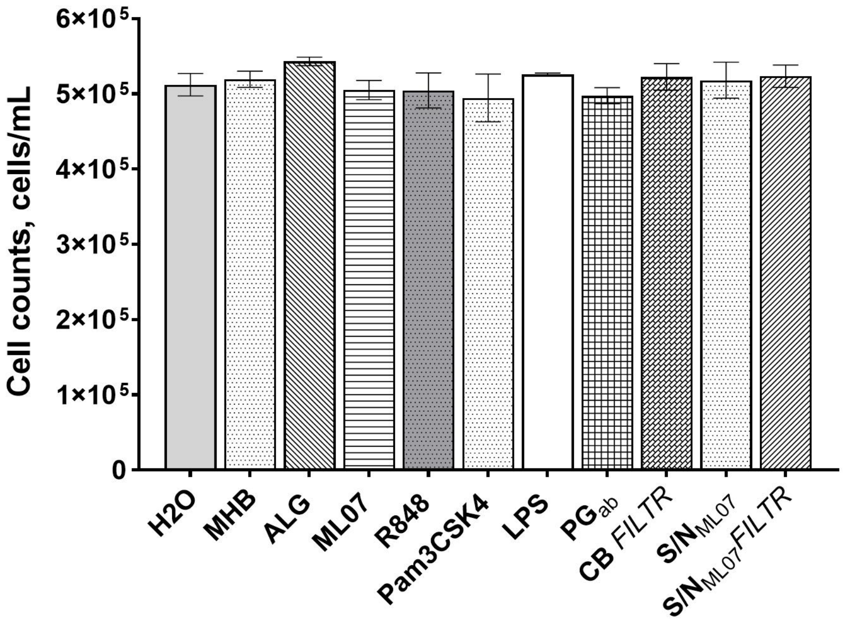

Figure S1. The amount of alive THP-1-Dual™ monocytes after the incubation with tested compounds: MHB (NC) - Mueller-Hinton broth sterile; Alg - Sodium alginate, 1%; ML07 - GRC-ML07; R848 (PC) - TLR7/8 ligand; Pam3CSK4 (PC) - Synthetic triacylated lipopeptide and a TLR2/TLR1 ligand; LPS (PC) - Purified lipopolysaccharide from *E. coli* strain 055:B5. TLR4 ligand; CB *FILTR* - Filter-sterilized culture broth after intact *P. aeruginosa* cells cultivation; S/N$_{ML07}$ - Supernatant of *P. aeruginosa* after the treatment with 1 mg/ml of GRC-ML07; S/N$_{ML07}$ *FILTR* - Filter-sterilized supernatant of *P. aeruginosa* after the treatment with 1 mg/ml of GRC-ML07. Data represent mean ± SD of three independent experiments.
